# Psychopathological symptoms as clinical phenotypes in suicide attempters: relation in terms of suicidal ideation, suicidal related behaviors and medical damage of the attempt

**DOI:** 10.1192/j.eurpsy.2023.1181

**Published:** 2023-07-19

**Authors:** D. Saiz-Gonzalez, P. Diaz-Carracedo, A. Pemau, W. Ayad-Ahmed, F.-R. Veronica, M. Navas Tejedor, A. de la Torre-Luque, M. Diaz-Marsa

**Affiliations:** 1 Psychiatry, Clinico San Carlos Hospital; 2 Psychiatry, Complutense University, Madrid, Spain; 3 Psychiatry, Complutense University, Madrid; 4 Psychiatry, CIBERSAM ISCII, Madrid, Spain

## Abstract

**Introduction:**

Suicide behaviour is a complex and multifactor concept that includes different risk factors. According with literature a dimensional concept of illness could help to understand this complexity and clarify clinical aspects of suicide risk.

**Objectives:**

The aim of this study is to identify different profiles of symptoms in a sample of suicide attempters and the relationship between this profiles and suicide behaviour in terms of outcome: presence and intensity of suicidal ideation, presence and number of attempts and severity of the medical damage in the current attempt.

**Methods:**

634 patients were recruited at the psychiatry emergency of eight public general hospitals in Spain between November 2020 until February 2022 in the SURVIVE protocol. The patients were assessed in 15 days using a battery of clinical tools that includes Brief Symptom Inventory, a sociodemographic interview, Mini Clinical Interview and C-SSRS, ACSS and BIS-11 scales. Latent profile analysis was applied to obtain profile symptoms. Logistic and multivariant regression was used to obtain data about outcome.

**Results:**

Three clinical profiles of psychiatric symptoms were described in suicide attempters (p < 0.01): high symptoms (HS) (45.02%), moderate symptoms (MS) (42.5 %) and low symptoms (LS) (12.48%). Significant differences were found between classes in four symptom domains (Figure 1): anxiety, obsessive-compulsive, sensitivity, and somatization (p < 0.01). Participants of the HS class showed higher values in relation with the BSI summary indexes, and more diagnoses, higher levels of suicidal ideation and suicidal related behaviour as well as higher acquired capability for suicide. Participants of the LS class were more likely to be women, older and unemployed and was related, according the analysis, with severe medical damage when compared with other groups (P< 0.01).

**Image:**

**Figure:**
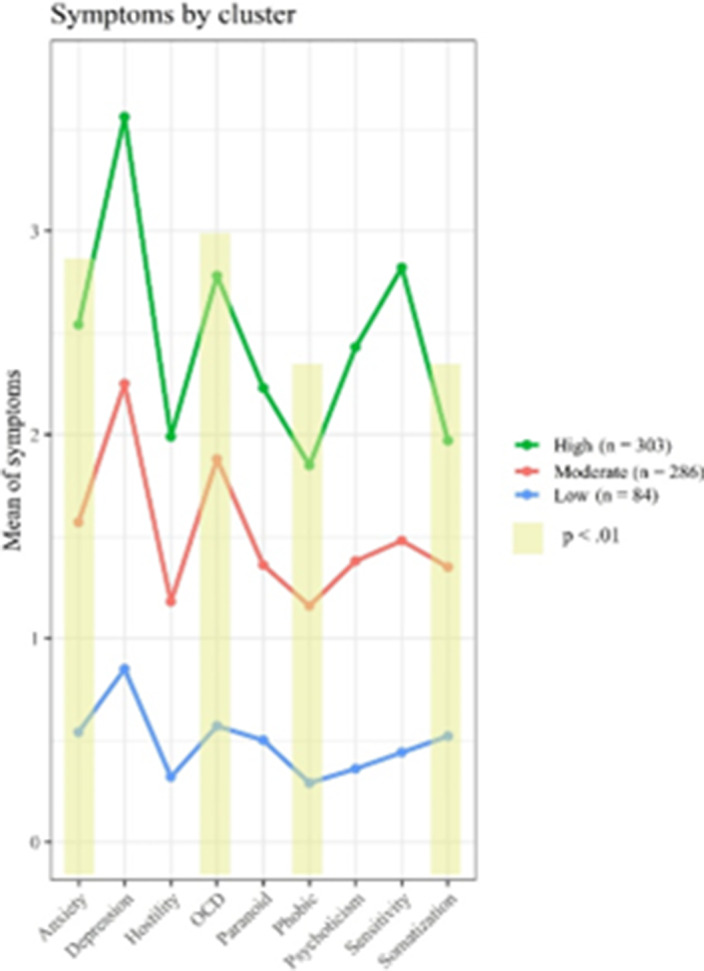

**Conclusions:**

According with the predictive model the study suggests different symptom-frequency clusters related with suicide attempt outcomes. Suicide ideation presence and intensity is related with HS class and acquired capability of suicide. Suicide ideation intensity is also related with number of diagnosis and number of previous attempts. Suicide behaviours presence is associated with being student and number with HS profile. Both presence and number were related with number of diagnosis as well as number of previous attempts (the higher all these clinical factors, the more intense of ideation in the last month). Finally, the severity of medical damage was related with LS profile and unemployed/retired work status. The dimensional symptom profile could be useful to predict suicide attempt outcome. Further study is needed to clarify this relation.

**Disclosure of Interest:**

None Declared

